# Mr. Hugo, on Vaccination

**Published:** 1807-04

**Authors:** Thomas Hugo

**Affiliations:** Crediton


### *To the Editors of the Medical and Phyſical Journal.*

GENTLEMEN,

THE following instance of the failure of Vaccination, in preventing the Small-Pox, is, I believe, the only one which has been recorded, as having occurred after it had been ascertained that the vaccine virus had acted *constitutionally*, and was not confined to the local vesicle on the arm.

It is, I think, fair to presume, that many of the failures which have been published, have arisen from the vaccine action not having extended beyond the inflammation of the inoculated part; as we observe that failures sometimes occur, from the same cause, in the variolous inoculation. In some of the reported cases of small-pox happening a second time, it seems, that in what is considered as the first occurrence of it, and on which the safety of the patient is supposed to depend, is merely the inflammation of the inoculated part, and the formation of a pustule, from which, perhaps, matter had been taken that communicated the disease to others: for there is frequently no mention of its having been succeeded by eruption, which, in the general mode of practice, is the only criterion of its febrile or constitutional action, and consequently of security against future infection. Indeed such repeated instances have passed under my own observation, of casual small-pox, where I had known that in a previous inoculation the arm had inflamed largely, but without its being succeeded by fever and eruption; that I now always think it prudent, under these circumstances, to caution my patients against exposure to variolous contagion, till they have, at some distant time, submitted to the test of another inoculation.—If the inflammation of the arm then, and the formation of a variolous pustule *only*, be considered as passing the small-pox, it is possible to have it not only twice, but, in certain habits, as often as a person will submit to be inoculated. It is well known, however, that neither the inflamed arm, nor the formation of the pustule, but that it is the *variolous fever* alone, which constitutes the security: and this, I believe, occurs so very rarely a second time in the same subject, that the proof of its existence must depend on very strong evidence indeed. In what proportion the second occurrence of variolous fever bears to cases of small-pox, after com-

 plete plete *constitutional* vaccination, is a most important subject for inquiry, as determining the degree of confidence to be placed in each.

In my superintendance of both these disorders, I have endeavoured to be equally careful to mark the proofs of their extending beyond the local inflammation of the inoculated part, and especially to watch the *vaccine* vesicle through its whole course, for the purpose of ascertaining the regularity of its appearance. For several years past, I have likewise in every regular case, made a second inoculation during the progress of the first, as a test of constitutional affection, and have never, during that period, vaccinated with any lymph, except such as I had obtained from some of the officers of the Jennerian Institution, in the first instance, or afterwards taken from a vesicle in the earliest stage possible, always desisting after an areola was formed.—In general also, I have used the further precaution of inserting it at *two distant* punctures; the one to supply me with lymph, for the purpose of vaccination, that I might leave the other untouched, to exert its influence on the system.

Having offered these observations as material to the credit of the following case, I shall proceed to state, that on account of the small-pox being prevalent in this neighbourhood, I inoculated, in November, 1803, the infant child of Mr. Woodman, aged only a fortnight, with recent vaccine lymph, making one puncture only. A vesicle formed, and went through its stages in the most regular manner, and during the progress of it I inoculated, as usual, the other arm. An areola formed round each puncture at the same time, proving very decidedly the constitutional affection. The vesicle from the second inoculation, preserved throughout its diminutive character, and the cicatrices of both now remain on the arms, different in size, but of the most regular appearance. From all these circumstances, I believe there can be no doubt of its having been *perfect* constitutional vaccination.

This opinion is also further strengthened, because several others were vaccinated with the same lymph as this child, and some from lymph obtained from this child's arm, who resisted, at that period, the contagion of the small-pox, and have since been inoculated with variolous matter without effect.—During the vaccination, however, this infant, on account of its early age, was not carried out of the house, and, I am informed, was not otherwise exposed at that time to infection.

The

The small-pox is now again become very general in this neighbourhood; and in compliance with the wishes of the parents, I have inoculated with variolous matter, a large proportion of those children whom I had, at different times before, vaccinated. In nearly the whole number, a small pustule only was excited on the arm, which dried off at the end of a week, without exciting any disorder of the system. In two or three cases, the patients became slightly feverish, and in one, was produced a considerable degree of fever, which continued for several days, accompanied with an universal rash; but not succeeded by eruption.

From the increased confidence which these trials produced in my mind, I thought it unnecessary to extend them farther, and gave that opinion to Mr. Woodman, on his application to me to inoculate his child: the event of which has proved the *only* exception to it, that has come within my observation. On inoculating this child, the inflammation of the puncture gradually went on in the usual manner, but instead of lessening about the eighth day, a violent accession of fever took place, and continued for three days, attended with an universal rash, and followed by about thirty variolous eruptions; only a few of them became pustular, and from these, matter was taken, which communicated the small-pox to two other children.

In this case, when we consider the precautions I had observed, and particularly that of having proved the existence of the constitutional affection, the vaccination must, I think, be admitted as complete: and the result of the subsequent inoculation is equally decisive of its failure, in this instance, as a security against the small-pox. I must repeat, however, that, as far as my observation has extended, it is a *solitary* exception, and I think there is, even here, some reason to believe that the variolous eruption was considerably lessened, though not wholly prevented, by the previous vaccination; as the number of the pustules bore no comparison to the great degree of febrile action which preceded. Yet we cannot, on the other hand but admit, that instances of trifling eruption, succeeding considerable fever, do often occur in variolous inoculation, where the necessary regulations with regard to diet and the mercurial regimen have been attended to: both which were particularly observed in this case.

I present it therefore to you, as a rare exception to the extraordinary security which vaccination generally affords against the contagion of small-pox. I am not influenced

 by by prejudice, nor have I a wish to lessen the merit due to so wonderful a discovery, no one having practised it with more attention, nor recommended it with more zeal and confidence than myself, but by the consideration that it is only from a knowledge of the unsuccessful, as well as of the favourable cases, that its real importance can be known, and the dependance to be placed in it, ascertained.

I am, &c.
THOMAS HUGO.

*Crediton, March* 16, 1807.

